# Curriculum Learning in Humans and Neural Networks

**DOI:** 10.1162/OPMI.a.355

**Published:** 2026-05-29

**Authors:** Younes Strittmatter, Stefano Sarao Mannelli, Miguel Ruiz-Garcia, Sebastian Musslick, Markus Wolfgang Hermann Spitzer

**Affiliations:** Department of Psychology, Princeton University, Princeton, NJ, USA; Data Science and AI, Computer Science and Engineering, Chalmers University of Technology and University of Gothenburg, Gothenburg, Sweden; School of Computer Science and Applied Mathematics, University of the Witwatersrand, Johannesburg, South Africa; Departamento de Estructura de la Materia, Física Térmica y Electrónica, Universidad Complutense de Madrid, Madrid, Spain; Grupo Interdisciplinar de Sistemas Complejos, Universidad Complutense de Madrid, Madrid, Spain; Institute of Cognitive Science, Osnabrück University, Osnabrück, Germany; Department of Cognitive and Psychological Sciences, Brown University, Providence, RI, USA; Department of Psychology, Martin Luther University Halle-Wittenberg, Halle, Germany

**Keywords:** curriculum learning, perceptual decision-making, parsimonious neural networks

## Abstract

The sequencing of training trials can significantly influence learning outcomes in humans and neural networks. However, studies comparing the effects of training curricula between the two have typically focused on the acquisition of multiple tasks. Here, we investigate curriculum learning in a single perceptual decision-making task, examining whether the behavior of a parsimonious network trained on different curricula would be replicated in human participants (*n* = 200). Our results show that progressively increasing task difficulty during training facilitates learning compared to training at a fixed level of difficulty or at random. Furthermore, a sequence designed to hamper learning in a parsimonious neural network network also impaired learning in humans. As such, our findings indicate qualitative similarities between neural networks and humans in curriculum learning for perceptual decision-making, suggesting the former can serve as a viable computational model of the latter.

## INTRODUCTION

*Curriculum learning* is the structured organization of training data or experiences, where the ordering follows a predefined strategy to influence the learning process. The effects of curriculum learning have been studied in both humans and neural networks (for humans, see Ahissar & Hochstein, 1997; Church et al., 2013; Liu et al., 2008; McLaren & Suret, 2000; Roads et al., 2018; for neural networks, see Hocker et al., 2024; Lee et al., 2024; Makino, 2023; Mannelli et al., 2024; Saglietti et al., 2022). These studies suggest that progressively increasing task difficulty typically facilitates learning. However, the relationship between curriculum learning in humans and networks remains unclear—mainly whether networks can serve as suitable models for human learning and whether human-inspired curricula can improve network training. Identifying similarities and differences may help to provide a deeper understanding of human cognition by examining it through neural network architectures. Conversely, these comparisons can inform the design of networks to more closely mimic human cognition (Dekker et al., 2022; Flesch et al., 2018; Lake & Baroni, 2023). However, despite the application of curriculum learning in both (e.g., Anderson et al., 1995; Wang et al., 2022), a direct comparison of curriculum learning between humans and networks in qualitatively similar tasks remains largely unexplored—especially in the context of single-task learning. Here, we systematically investigate the effects of different curricula during perceptual learning in humans and a neural network.

Prior studies comparing human and neural network learning have focused on their similarities and differences concerning sequential acquisition of multiple tasks. For example, Flesch et al. (2018) investigated *continual learning*—learning multiple tasks in sequence—in humans and networks. Their behavioral findings suggest that humans learn categorization tasks more effectively when training trials are grouped into task blocks instead of interleaving tasks. In contrast, their simulations indicate that networks perform better when interleaving tasks and that they suffer catastrophic forgetting when training trials are blocked. Similarly, Dekker et al. (2022) observed that humans learn new tasks remarkably quickly by generalizing knowledge to new settings, while standard networks fail to do so. However, the authors also reported that more advanced neural networks—especially those trained with structured learning approaches—can achieve human-like performance (for another example, see Lake & Baroni, 2023). While these studies have mainly highlighted differences in learning between humans and neural networks, they have primarily focused on the sequential acquisition of multiple tasks.

In contrast, studies considering perceptual learning typically examined training curricula within a single task. For instance, Church et al. (2013) demonstrated that humans performed better on a hard auditory perceptual discrimination task when previously trained on trials with progressively increasing difficulty compared to trials with decreasing or consistently hard difficulty throughout the training. Similarly, Mannelli et al. (2024) found that parsimonious neural networks learned a perceptual discrimination task more efficiently when trained with progressively increasing difficulty rather than consistently hard or random difficulty.^1^

Studies on curriculum learning typically use training regimes that include *ascending*, *hard*, or *random*^2^ curricula. After training, researchers generally assess the performance in a testing phase consisting exclusively of hard trials. (e.g., Church et al., 2013; Mannelli et al., 2024). An ascending curriculum structures learning by gradually increasing the task difficulty, while a hard curriculum examines an alternative hypothesis that training on trials with consistently hard difficulty, matching that used in testing, maximizes learning efficiency. Random difficulty curricula serve to control for the alternative account that not progressively increasing difficulty but rather variability in difficulty maximizes learning efficiency. Comparisons between these conditions help determine whether progressively increasing task difficulty uniquely enhances learning or if alternative approaches yield similar results.

### The Present Study

**Figure 1.**
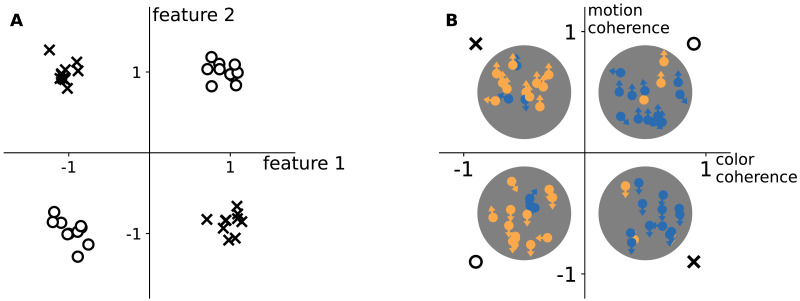
Discrimination task for neural networks (A) and humans (B). For neural networks (A), stimuli are sampled from four isotropic Gaussian centered in ±*μ*_0_, ±*μ*_1_ ∈ ℝ^*d*^, with *μ*_0_ and *μ*_1_ orthogonal. The figure shows a low-dimensional projection that illustrates with shapes the class labels. Labels follow the XOR rule: diagonally opposite quadrants share the same label. Task difficulty is controlled by the Gaussian standard deviation *σ*. For humans (B), the same XOR structure is implemented in a perceptual space defined by motion and color. For example, “yellow-up” and “blue-down” required the same response, while “yellow-down” and “blue-up” required a different response. Motion coherence is the fraction of dots moving in a signal direction (others move randomly), where +1 corresponds to 100% upward motion and −1 to 100% downward motion. Color coherence is the fraction of dots in the dominant color, where +1 corresponds to 100% blue and −1 to 100% yellow.

In this study, we investigated curriculum learning in a perceptual decision-making task for both humans and parsimonious neural networks. Inspired by the modeling work of Mannelli et al. (2024), we designed corresponding tasks for both a neural network and humans, each requiring integration across two feature dimensions, with the added complexity of noisy feature values in both dimensions. Crucially, we structured the tasks so that the features had to be integrated following an XOR rule (see Figure 1). This design allowed us to examine curriculum learning by manipulating feature noise (e.g., increasing noise over time in an ascending curriculum). More importantly, this design also allow for a bad curriculum through blocked learning, where feature pairings were systematically grouped, restricting exposure to certain feature combinations.

## CURRICULUM LEARNING IN NEURAL NETWORKS

To examine the similarity between human and neural network perceptual learning, we conducted numerical experiments to determine whether networks’ predictions generalize to human behavior.

Specifically, we compared how four training curricula affect the accuracy of the same network architecture with different weights initializations. Based on previous theory on the beneficial effects of an ascending curriculum (e.g., Mannelli et al., 2024), we expected networks trained on an ascending curriculum to outperform a random and hard curriculum. This also allowed us to design a “bad curriculum” whose objective was to disrupt performance in the network–see the Stimuli section below.

In addition to examining performance on test trials, we analyzed the training trajectory. We split the neural network population into *high-achieving* networks exceeding 65% accuracy and *low-achieving* networks falling below this threshold. This allowed us to examine whether overall performance differences were due to curricula affecting all networks similarly—essentially shifting the entire performance distribution while maintaining its shape—or whether curricula altered the distribution itself, impacting the proportion of high-achieving networks. Finally, as an additional exploratory analysis, we examined the long-term impact of initial training conditions by exposing neural networks to extended training following the initial curriculum training.

### Methods

#### Architecture.

Similar to Mannelli et al. (2024), the neural networks consisted of two layers with *K* = 4 hidden units and a ReLU activation function. We trained these networks using binary cross-entropy loss and online stochastic gradient descent updates. With *K* = 4, the network is capable of parsimoniously solving an XOR task in its optimal weight configuration (Ben Arous et al., 2024; Mannelli et al., 2024; Refinetti et al., 2021). We accounted for individual variability by initializing the network weights drawn from a Gaussian distribution with a mean of 0 and a standard deviation of 0.05.

#### Stimuli.

As the perceptual discrimination task (Figure 1A), we used an *XOR Gaussian-Mixture model* (Refinetti et al., 2021), where inputs are sampled from four Gaussian distributions whose means (±*μ*_*y*_) are arranged to form an XOR, and the XOR rule determines the labels. Formally, given a label from *y* ∼ Bern($\frac{1}{2}$), *x* ∼ $\frac{1}{2}$𝒩(*μ*_*y*_, *σ*^2^𝕀_*d*_) + $\frac{1}{2}$𝒩(−*μ*_*y*_, *σ*^2^𝕀_*d*_), with *μ*_0_, *μ*_1_ ∈ ℝ^*d*^ orthogonal unit vectors. Large standard deviation (*σ*) creates less distinct Gaussians, making the task harder.

We compared four training curricula—ascending, hard, random, and bad—and assessed accuracy in a testing phase of exclusively hard trials. In the training trials, the standard deviation (and thus the difficulty levels) ranged from *σ* = 0.1 (easy) to *σ* = 0.65 (hard), except for the hard curriculum where we fixed *σ* at 0.65. For the ascending curriculum, *σ* linearly increases to increase difficulty. We used the same difficulty levels as the ascending curriculum in the random curriculum but presented them in a randomized order. In the bad curriculum, we presented trials in random order of difficulty, but we manipulated the presentation to show only a subset of features (context-stimulus pairs) ±1 at the beginning and the other subset ±1 at the end.^3^ Throughout training the probability of observing the initial subset linearly decreased, while the probability of observing the other increased. This design limited early exposure to the complete set of feature pairings, making it harder to generalize the XOR-based response strategy across the task.

#### Procedure.

Building on the work of previous studies (cf. Ben Arous et al., 2024; Mannelli et al., 2024; Refinetti et al., 2021), we employed a mean-field approach from statistical physics to characterize the learning dynamics. At an intuitive level, the mean-field approach can be understood as describing the average learning behavior of a large population of similar networks, rather than the noisy trajectory of any single network. Instead of following individual weight updates, it tracks how groups of weights tend to align with task-relevant features over time. This allows learning to be interpreted as a smooth trajectory through a low-dimensional space of representations, where different training curricula bias the system toward different learning paths early on. In this sense, curricula do not change what the network can learn in principle, but they strongly influence which solution the network commits to during learning.

This approach reduced the stochasticity of learning to the initialization phase and allowed us to analytically evaluate the learning trajectory without requiring numerical simulations. This provided a fast and precise characterization of the learning process. After training for 10 time units with a learning rate of 10, where time in the mean-field approach is given by epoch/input dimension, we evaluated the accuracy exactly using the mean-field solution of the problem. We ran 10,000 simulations for each curriculum to characterize the accuracy distribution. To explore long-term impact, we trained the neural networks on additional hard trials up to 100, 1,000, and 10,000 time units after the initial 10 time units.

In this framework, time is measured in normalized time units, defined as training epochs scaled by input dimensionality. This normalization allows learning progress to be compared across different settings and emphasizes that early training corresponds to a regime in which representations are still highly malleable. As learning proceeds, weight alignments stabilize, and the system becomes increasingly constrained in how easily it can revise its internal representation.

#### Data Analysis.

Given this large sample size of 10,000 for each condition, we used Cohen's *d* to assess statistically significant differences between curricula.

### Results

#### Testing.

**Figure 2.**
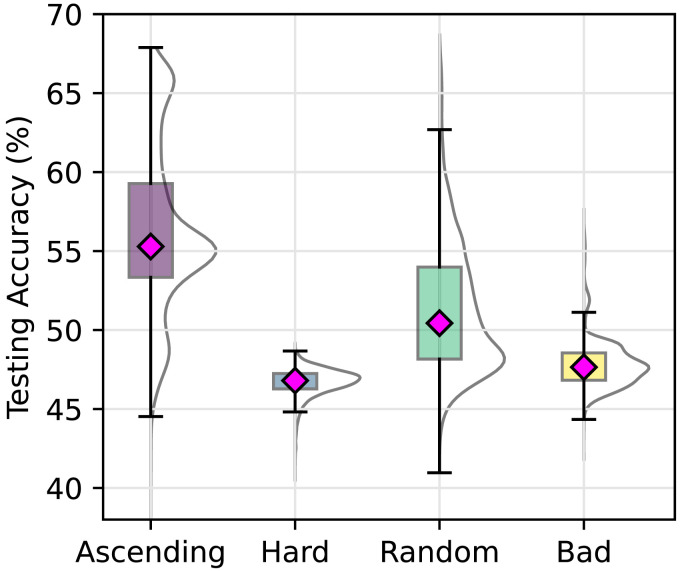
Boxplots showing the testing accuracy of neural networks after training under four different curricula. Each violin plot shows the distribution of test accuracy across 10,000 network simulations for the ascending, hard, random, and bad curricula. The ascending curriculum yields the highest accuracy, while the hard, random, and bad curricula result in significantly lower performance. Magenta diamonds indicate the mean testing accuracy for each curriculum. Boxes indicate the middle 50% of the distribution. The full distribution of network accuracies is illustrated by the half-violins.

Figure 2 depicts the accuracy of neural networks in test trials. The ascending curriculum showed on average the highest accuracy, and reported a large and statistically significant effect against the other curricula. Specifically, the absolute value of Cohen’s *d* was 0.98 for the ascending curriculum against the random curriculum, 2.5 against the hard curriculum, and 2.1 against the bad curriculum. The random curriculum also demonstrated a large and statistically significant effect size, with values of 1.5 against the hard curriculum and 1.1 against the bad curriculum, performing better than the hard and bad curriculum but worse than ascending. The smallest, yet significant, effect size of 0.90 was observed between the hard and bad curricula, indicating relatively higher accuracy for the bad curriculum.^4^

#### Training.

**Figure 3.**
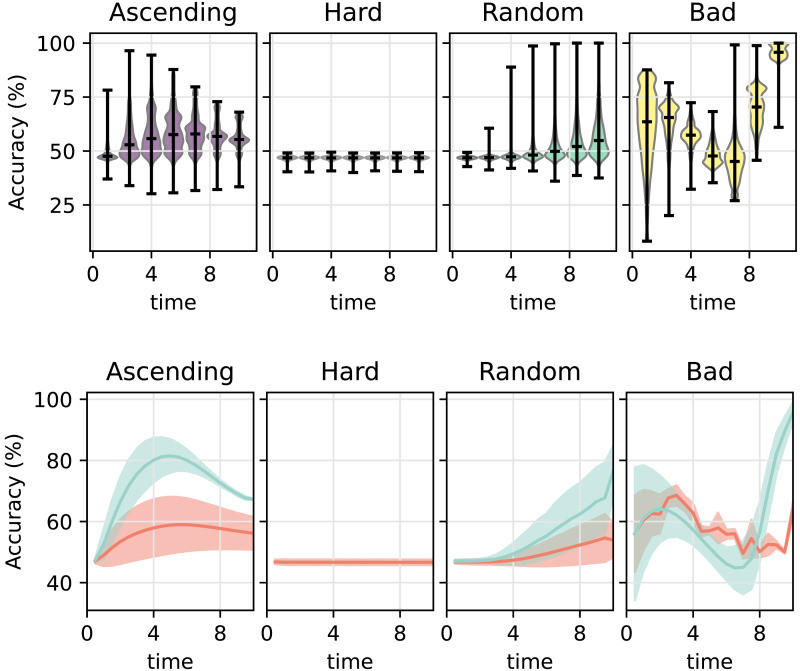
Training accuracy of neural networks under four curricula. The upper panel shows the distribution of accuracy across networks at each training time unit, illustrating how performance evolves over training. While some curricula—particularly the bad curriculum—produce high apparent training accuracy, this does not necessarily reflect successful learning of the full XOR structure. Vertical bars indicate the average training accuracy. The full distribution of network accuracies is illustrated by the violins. The lower panel shows the same training trajectories separated into networks that achieved above (teal) or below (red) 65% accuracy in the final training time unit, revealing distinct learning outcomes across initializations. Under the ascending curriculum, a subset of networks converges to high-performing solutions, whereas under the hard curriculum networks remain near chance. The random and bad curricula produce heterogeneous outcomes, with some networks achieving high training accuracy while others fail to learn the full task. Solid lines indicate mean accuracy and shaded regions indicate ±1 standard deviation.

The upper panel of Figure 3 depicts the accuracy of neural networks in training trials, while the lower panel depicts the training trajectories when splitting the networks into a high-achieving group (above 65% accuracy) and low-achieving group (below 65%) based on the accuracy of the final training time unit. Since trial difficulty varied between curricula in the training but not in the test trials, we also report the proportion of networks above threshold performance: In the ascending curriculum, 11.62% of networks performed above the threshold in the final training trials, and 11.46% did so in the test trials. In contrast, none of the networks trained with the hard curriculum surpassed the threshold in either training or test trials. For the random curriculum, 18.96% of networks exceeded the threshold in the final training trials but only 1.07% in the test trials. In the bad curriculum, 99.97% of networks surpassed the threshold in the final training trials, yet none did so in the test trials.

#### Long-Term Impact.

**Figure 4.**
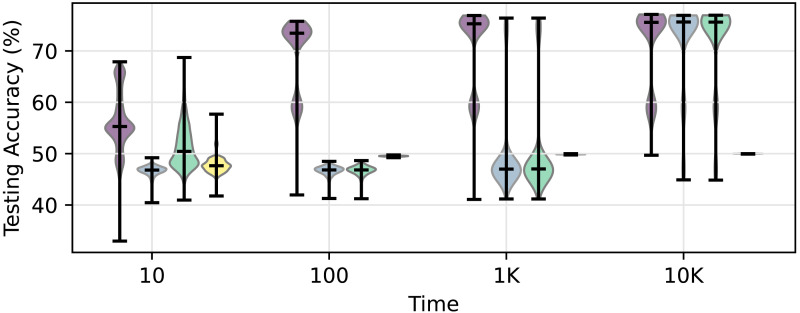
Testing accuracy after additional hard training following initial curriculum exposure. Each group of violin plots shows the distribution of test accuracy across network initializations after the initial curriculum training (10 time units), followed by additional hard training for 100, 1K, or 10K time units. Networks trained under the ascending curriculum maintain high performance throughout. Networks trained under the hard and random curricula initially perform poorly but improve substantially with extended training, eventually reaching similar accuracy levels as the ascending curriculum. In contrast, networks trained under the bad curriculum fail to recover even after prolonged additional training, indicating persistent convergence to incomplete task representations. Violins represent the distribution of the networks and vertical bars correspond to the average accuracy. Note, the first group of violin plots reproduces the results shown in Figure 2.

Figure 4 depicts the accuracy in additional hard training trials followed by an initial curriculum training phase. While continued training on to 100 trials following an initial ascending curriculum further improves accuracy, networks initially trained with a hard, random, or bad curriculum do not show accuracy gains. However, while this trend persists for 1,000 trials, after 10,000 trials, networks initially trained with a hard or random curriculum eventually catch up and reach similar accuracy levels to networks trained on an ascending curriculum. In contrast, networks initially trained with a bad curriculum continue to underperform, failing to reach accuracy above chance even after receiving trials.

### Discussion: Neural Networks

Altogether, the neural network simulations suggest that training with an ascending curriculum is the most effective approach for learning a perceptual decision-making task.

Our analysis of training trajectories and the split between high- and low-achieving networks in the test phase shows that ascending curricula increase the proportion of high-achieving networks compared to the other conditions. These findings align with prior studies; for instance, Mannelli et al. (2024) demonstrated that an ascending curriculum expands the proportion of networks that lead to high performance.

Furthermore, our analysis identifies discrepancies between the proportion of high-achieving networks at the end of training and in the test phase for the random and bad curricula, whereas such discrepancies are absent in the ascending and hard curricula. This effect is particularly pronounced in the bad curriculum. These findings provide insight into the underlying mechanisms determining whether networks successfully acquire the task.

The results of the long-term impact analysis indicate that for the chosen difficulty level the network can successfully learn the XOR task when provided with sufficient training data, even under the hard curriculum. However, the detrimental effects of exposure to the bad curriculum appear irreversible, suggesting that the network becomes trapped in an attractor state induced by learning only one subset of the task, preventing successful generalization.

## CURRICULUM LEARNING IN HUMAN PARTICIPANTS

Like the neural network simulations, we conducted a between-group web-based experiment to examine whether the same four training curricula affected participants’ accuracy on subsequent hard test trials. The test trials were identical across training curricula. Based on the neural network simulations, we expected the ascending group to outperform the hard and random groups, as reflected in significantly higher accuracy. Additionally, we anticipated higher accuracy in the random group compared to the hard group during testing, also based on the network simulations. Regarding the bad curriculum, although previous empirical work suggests that blocked learning benefits learning in humans (Flesch et al., 2018), our network simulations predicted lower accuracy for this group, leading us to expect weaker performance compared to the other conditions.

In addition to examining test performance, we analyzed the training trajectory of human participants, similar to our approach with neural networks. We also categorized participants into high-achieving—above 65% accuracy–and low-achieving—below 65%—groups to investigate whether overall performance differences were primarily driven by curricula influencing all individuals similarly or whether curricula instead affected the distribution of participants who performed well versus poorly.

### Methods

#### Participants.

We collected data from 200 participants (mean age = 23.1) through an online experiment administered on Prolific. We implemented within-counterbalancing using SweetPea (Musslick et al., 2022) and automated the experiment execution via AutoRA (Musslick et al., 2024). All participants were between 18 and 45 years old and consented to participate. The experiment lasted five minutes on average. The data was partially collected at Brown University, Providence, USA, where the study received approval from the Institutional Review Board, and at Martin-Luther University, Halle-Wittenberg, Germany, where the study was also in accordance with ethical guidelines.

#### Stimuli.

We exposed participants to a perceptual decision-making task (see Figure 1B) in which they had to identify the majority motion direction or majority color of dots in a random-dot-motion kinematograms (RDK). The task followed an XOR rule, meaning responses depended on the conjunction of these features—for example, “yellow-up“ and “blue-down“ required the same response, while “yellow-down“ and “blue-up“ required a different response. We used ‘F’ and ‘J’ as response keys and instructed participants to use the left and right index fingers. The key-response mapping was counterbalanced between participants.

We administered RDK trials using the rdk-plugin (Rajananda et al., 2018) and always presented 600 dots during both training and testing. The dot radius was set to 2 pixels, and the moving distance was set to 1 pixel per frame. To manipulate task difficulty during training, motion coherence ranged from 40% to 100%, indicating the percentage of the dots moving coherently in the target direction instead of in random directions. The color coherence ranged from 66% to 100%, indicating the percentage of dots colored with the target color as opposed to the opposite color. Notably, color coherence was not systematically manipulated to create curricula but counterbalanced across trials. We chose this task as coherence decreases typically affect humans’ performance in the task, leading to increased error rates and thus increasing the difficulty of the task (Baker et al., 1991; Lankheet & Verstraten, 1995; Strittmatter et al., 2023, 2024).

The difference between the four training curricula was the sequence in which the trials were presented. Similar to the noise in the neural networks training, we manipulated the sequence of motion coherence in the ascending, hard, and random curricula. In the ascending curriculum, we decreased the motion coherence linearly from 100% to 40% coherence. For the hard curriculum, we set the coherence to 40% throughout the trials, and in the random curriculum, the motion coherence was the same as in the ascending but randomly ordered. In the bad curriculum, the trials again had the same motion coherence as in the ascending condition, but the feature pairing was blocked. For example, with the response-key mapping “yellow-up“ and “blue-down“ to ‘F’ and “yellow-down“ and “blue-up“ to ‘J’, the first half of trials contained the pairings “yellow-up“ and “yellow-down“ (or “yellow-up“ and “blue-up“), while the second half contained the pairings “blue-up“ and “blue-down“ (or “yellow-down“ and “blue-down“, respectively). Thus keeping one feature dimension constant in each subset parallel to the procedure described for the neural networks. All possible feature subsets and response-key mapping were counterbalanced between participants. Additionally, as in the training of the neural network, the proportion of trials from one subset decreased, while the proportion of trials from the other subset correspondingly increased over the training.

#### Procedure.

We randomly assigned participants to four different training curricula, with 50 participants per curriculum, while all participants responded to the same test trials. We instructed them to perform the task as accurately as possible on each trial and that they could gain a bonus of 0.02$ dollar per trial for accurate performance during the test trials. Next, the participants had to respond to the colored RDKs. Importantly, the experiment comprised two phases: a training phase and a testing phase. We presented 100 training trials and 16 test trials to participants.

#### Data Analysis.

We conducted the data analysis in R (R Core Team, 2021). We first evaluated whether the different training curricula affected participants’ accuracy and RT. The raw data and data analysis scripts are available on https://osf.io/p5rgx/.

To estimate differences in accuracy between curricula during testing, we ran a hierarchical logistic regression model using the lme4 package (Bates et al., 2015), with the factor curricula as the independent variable and participants’ accuracy as the dependent variable. A random intercept was applied to control for the overall variability in accuracy between the participants. We did not apply a random slope term as the model with a random slope term did not converge. We used the *emmeans* package to evaluate the pairwise comparisons between all curricula (Lenth et al., 2018). Finally, and as with the neural network simulations, we illustrated participants’ performance trajectory on their training sequences.

### Results

#### Testing.

**Figure 5.**
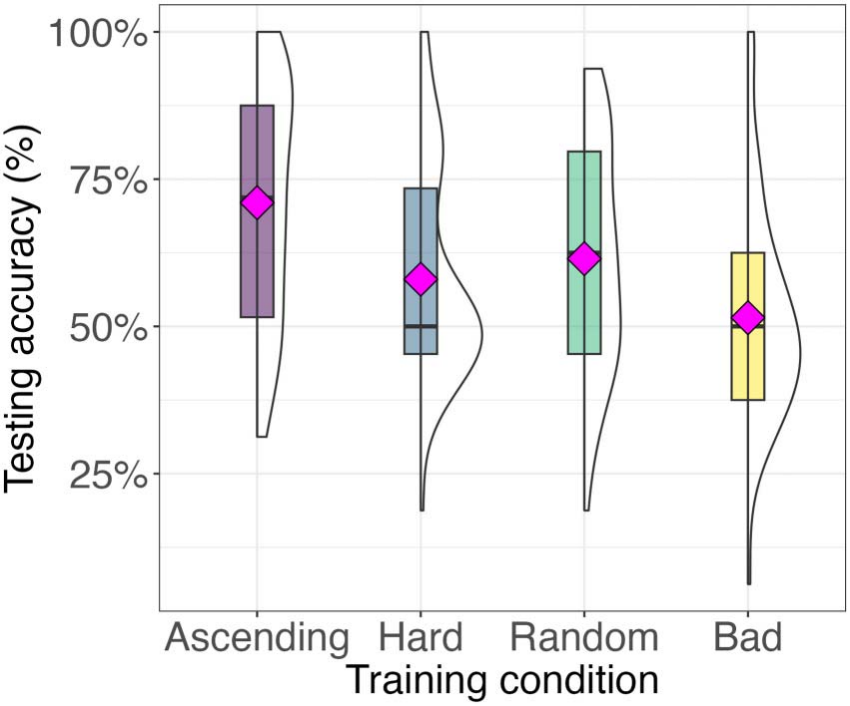
Boxplots showing accuracies on hard test trials for human participants previously training on four different curricula. The ascending curriculum yields the highest average test accuracy, while the hard, random, and bad curricula result in significantly lower accuracy. Boxes indicate the interquartile range (middle 50% of the distribution), horizontal lines indicate the median, and magenta diamonds indicate the mean accuracy.

Figure 5 depicts the accuracy of human participants in the test trials. We observed that participants were significantly more accurate during testing if they were previously exposed to an ascending curriculum, compared to a hard, random, or bad curriculum; ascending vs. hard: *beta* = .65; *z* = 3.58; *p* < .001; ascending vs. random: *beta* = .49; *z* = 2.69; *p* = .007; ascending vs. bad: *beta* = .857; *z* = 4.72; *p* < .001). Participants with the hard curriculum did not significantly differ in accuracy during testing compared to the random and to the bad curriculum (hard vs. random: *beta* = −.16; *z* = −.89; *p* = .373; hard vs. bad: *beta* = .21; *z* = 1.16; *p* = .244). Participants with the random curriculum achieved significantly higher accuracies during testing than the bad curriculum (random vs. bad: *beta* = .37; *z* = 2.05; *p* = .040).

#### Training.

**Figure 6.**
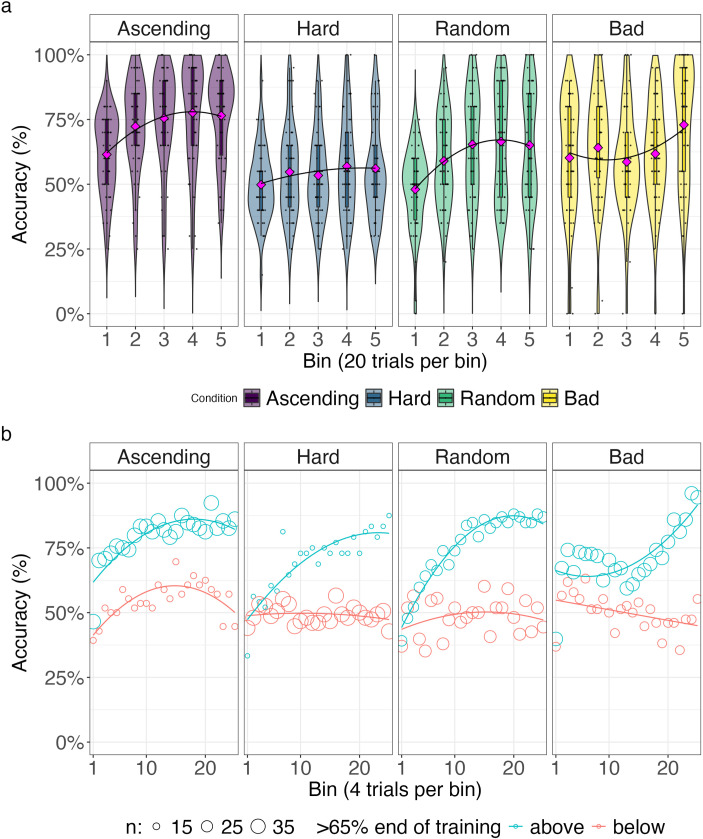
Training accuracy trajectories of human participants under four curricula. (a) Distribution of participants’ accuracy across training, shown in bins of 20 trials. (b) The same trajectories shown in finer resolution (average accuracy of 4 trials indicated as circles), separated into participants who achieved above (teal) or below (red) 65% accuracy in the final 20 training trials. Solid lines indicate smoothed averages, and circle size reflects the number of participants contributing to each circle.

Figure 6 depicts the accuracy of human participants in the training trials. Note, to reduce noise and calculate accuracy estimates on the participant level, we binned the training trials in bins of 20 trials to analyze the accuracy during training. The upper panel in Figure 6 illustrates the average accuracy for every bin and for each curriculum. Similar to the procedure for the neural networks, we then split the participants into a high-achieving group (above 65% accuracy) and a low-achieving group (below 65%). The lower panel of Figure 6 depicts the training trajectories when splitting the groups based on the accuracy of the final 20 training trials. Note, here we also report the proportion of participants that reached above threshold accuracy on test trials. Most participants in the ascending curriculum exceeded the accuracy threshold in the final 20 training trials (72% of the group) and testing (60%). In the hard curriculum, only 24% met the threshold during training and 30% during testing. In the random curriculum, 46% reached the threshold in training and 40% in testing. The bad curriculum had the second-highest training accuracy during the final 20 trials (63%) but the fewest participants who performed above the threshold during testing (16%).

### Discussion: Human Participants

The behavioral results from human participants qualitatively align with the findings from the neural network simulations. Notably, we not only replicated the superior performance of participants exposed to the ascending curriculum compared to those in the hard and random curricula but also observed similarly poor performance in the bad curriculum. This similarity extends beyond average trends, as both humans and networks exhibited comparable splits into high- and low-achieving groups.

As in the network simulations, discrepancies in the proportions of high-achieving participants between the final training trials and the test trials emerged in the bad curriculum but not in the ascending, hard, and random curriculum, demonstrating strikingly similar patterns. These findings suggest that the underlying mechanisms driving failure in the bad curriculum were comparable in humans and networks—specifically, participants performed well on one feature subset while failing to retain the other. However, it is important to note that, unlike in networks, we did not assess the long-term impact of training on humans. This remains an open question for future research.

## GENERAL DISCUSSION

We conducted this study to evaluate potential similarities and differences between neural networks and humans in curriculum learning when learning on a single task. Specifically, we first trained neural networks on four different curricula—ascending, hard, random, and bad—and assessed their performance during a subsequent hard testing phase. We then applied the same curricula to human participants to investigate whether similar patterns emerged.

Our results indicate that both humans and networks benefited from an ascending curriculum compared to the hard and random conditions. As expected, the bad curriculum, a blocked design meant to hinder learning for neural networks (cf. Flesch et al., 2018), led to poor network performance. Interestingly, contrary to previous studies suggesting that blocked learning benefits human learning (e.g., Flesch et al., 2018), in our task design, humans also performed poorly on this curriculum.

A key distinction between our study and previous research on blocked learning is the nature of the tasks being acquired. Prior studies typically examine the learning of multiple independent tasks or stimulus sets presented in a blocked fashion. In contrast, while our XOR task may superficially appear to involve learning two separate stimulus–response mappings, successful performance requires integrating both feature dimensions rather than treating them as independent units. This requirement helps explain why the structured benefits of blocked training, which typically facilitate the acquisition of distinct tasks, do not emerge here. In the bad curriculum, early training is dominated by a restricted subset of feature pairings, allowing participants to achieve high accuracy during training by relying on a partial solution that does not require integrating both dimensions according to the XOR rule. However, this incomplete representation fails when learners are later required to generalize to the full set of feature combinations during testing. This pattern closely mirrors the behavior of the neural networks, which under the bad curriculum converge to a solution that captures only part of the task structure and fail to learn the full XOR rule even after subsequent exposure to the remaining feature combinations. Together, these findings suggest that both humans and networks are vulnerable to early commitment to incomplete representations when training does not sufficiently encourage integration across task components. From this perspective, the similarity between humans and networks does not imply architectural equivalence, but reflects comparable constraints on learning dynamics, whereby early training structure can limit the ability to revise representations once learning has progressed.

Importantly, analyzing training performance in relation to test performance allowed us to generate new hypotheses regarding the factors that contribute to success or failure in both humans and neural networks. First, our findings indicate that differences in learning outcomes are at least partially driven by shifts in the proportion of high-achieving versus low-achieving learners, replicating results from Mannelli et al. (2024). Second, by examining discrepancies between training and test performance—most pronounced in the bad curriculum—we observed that humans exhibit similar patterns of catastrophic forgetting as neural networks.

However, while we demonstrated that the detrimental effects of the bad curriculum persist over time in neural networks, we did not conduct a corresponding long-term experiment for human participants. Investigating the long-term consequences of different curricula in humans therefore remains an important direction for future research. The neural network simulations show that networks trained under hard and random curricula can eventually recover and learn the full XOR structure, but only after very extensive additional training, whereas networks trained under the bad curriculum fail to recover even after prolonged exposure to hard trials. This suggests that the long-term effects of curricula depend not only on initial performance but also on whether learners can revise incomplete representations with sufficient additional experience. In the human data, the bad curriculum already produces a pronounced dissociation between training and test performance within the first 100 trials, consistent with early reliance on an incomplete representation of the task. However, it remains unclear whether human learners would similarly succeed if given substantially longer training on hard trials after the hard or random curriculum, or whether their performance would remain limited. Likewise, it is unknown whether introducing interleaved hard trials after blocked training could mitigate the negative impact of the bad curriculum. Addressing these questions would require extending training to several hundred trials, potentially across multiple sessions, while carefully accounting for fatigue and boredom effects that may arise during prolonged training and influence learning outcomes.

A notable limitation of this study is that, while we applied neural network-inspired curricula to human participants, we did not explicitly model the neural networks to align with human behavior. In both systems, ascending curricula facilitate learning, the bad curriculum leads to dissociations between training and test performance consistent with incomplete task representations, and curricula influence the distribution of successful versus unsuccessful learners. These shared qualitative features suggest that parsimonious neural networks capture key aspects of how learning dynamics depend on training order, even though humans and networks differ substantially in architecture, learning mechanisms, and sources of variability. At the same time, we acknowledge differences between the results observed for humans and networks. For example, variability in neural network performance tends to scale with mean performance across curricula, whereas human variability does not follow the same proportional relationship and remains similar across curricula. Additionally, we do not provide a mechanistic explanation for why either humans or networks exhibit the observed learning patterns. Mannelli et al. (2024) offers a starting point for such an investigation by demonstrating that the effects of curriculum learning emerge only in parsimonious neural networks and not in deep neural networks. Yet, describing the human brain as parsimonious is not biologically plausible, and the only inference we can confidently draw from these comparisons is that the brain must be subject to certain constraints^5^. However, further work is required to shed light into the similarities of parsimonious neural networks and human learning.

In addition, in the neural network simulations task difficulty is controlled by isotropic input noise, such that variability is present along both feature dimensions. In the human experiment, we structured the curriculum using motion coherence only, while color coherence was varied across trials but not systematically ordered. This design choice reflects the fact that motion coherence provides a robust and well-characterized manipulation of perceptual difficulty in humans, whereas structuring both dimensions simultaneously would have substantially increased task complexity and reduced interpretability. Crucially, the curriculum effects observed in both systems do not depend on symmetric variance across dimensions, but on the structure of early exposure to feature conjunctions. In both the neural network and human tasks, the bad curriculum restricts early access to the full set of XOR-relevant feature pairings, and it is this restriction—rather than the specific source of variability—that drives the observed learning failures. We note that we did not run a separate neural network control in which variability was structured along only a single input dimension; however, the qualitative curriculum effects we observe arise from restricted early exposure to feature conjunctions rather than from symmetric variance across dimensions.

Taken together, our findings reveal striking similarities in curriculum learning between humans and parsimonious neural networks. Our results also highlight promising directions for future research, particularly regarding the long-term implications of different curricula and the underlying mechanisms driving the observed learning effects. A deeper understanding of these mechanisms could have important implications for both educational curricula and the development of optimized training sequences for more efficient neural networks.

## ACKNOWLEDGMENTS

S.S.M. was supported by the Wallenberg AI, Autonomous Systems, and Software Program (WASP). S.M. and Y.S. were supported by the Carney BRAINSTORM program at Brown University. M.R.-G. acknowledges support from the Ramón y Cajal program (RYC2021-032055-I) and the Spanish Research Agency (PID2023-147067NB-I00). M.R.-G. and M.W.H.S. are supported by a Research Grant from HFSP (Ref.-No: RGEC33/2024; https://doi.org/10.52044/HFSP.RGEC332024.pc.gr.194170).

## AUTHOR CONTRIBUTIONS

Y.S.: Conceptualization; Methodology; Writing – original draft. S.S.M.: Conceptualization; Formal analysis; Writing – original draft. M.R.-G.: Conceptualization; Methodology; Writing – original draft. S.M.: Conceptualization; Methodology; Writing – original draft. M.W.H.M.: Conceptualization; Methodology; Writing – original draft.

## Notes

^1^ Curriculum learning did not significantly improve the training of a deep neural network in their study.^2^ Matching the difficulty trials of the ascending curriculum, presented in random or descending order.^3^ Our curriculum designs are based on well-informed heuristics from the neural network literature, particularly Cornacchia and Mossel (2023), Saglietti et al. (2022), and Mannelli et al. (2024). While recent theoretical advances by Mignacco and Mori (2025) and Mori et al. (2025) may now allow for the mathematical identification of best and worst curricula, this remains an active field of research. In our analysis, we used insights from Mannelli et al. (2024) to formulate our version of a bad curriculum; this differs from a descending curriculum, which has been analyzed in different models by Hacohen and Weinshall (2019) and Saglietti et al. (2022).^4^ The network returns three values: returning one of the two labels or zeroing out and not returning any label. This lowers chance-level accuracy by allowing the network to forgo an answer.^5^ We used the term constraints to refer to the difficulty both systems face in transitioning from a ‘simple’ perceptual solution to a ‘demanding’ rule-based solution. In our XOR-like task, the ‘simple’ solution involves responding based on isolated perceptual features, while the ‘demanding’ solution requires integrating these features according to a contextual rule. In human learners, we propose that this tension arises from the interplay between fast and slow learning systems, where a tendency to stabilize on simpler, internally consistent representations can limit the flexibility needed to restructure learned mappings. In the parsimonious neural networks studied here, a similar constraint emerges from gradient descent dynamics. The small number of hidden units creates an attractor structure where the network may commit to early, incomplete internal representations based on initial input statistics. Consequently, the ‘constraints’ we observe are functional: they represent a system’s sensitivity to early training and a restricted ability to revise learned structures once an adequate (but incomplete) solution is found. This shared dynamical bottleneck explains why both humans and networks benefit from an ascending curriculum that guides them toward the ‘demanding’ solution and why both are equally impaired by ‘bad’ curricula that trap them in the ‘simple’ one.
